# Molecular pathways in the development of HPV-induced oropharyngeal cancer

**DOI:** 10.1186/s12964-023-01365-0

**Published:** 2023-12-14

**Authors:** Muhammad Ikram Ullah, Maria V. Mikhailova, Ali G. Alkhathami, Nestor Cuba Carbajal, Manuel Enrique Chenet Zuta, Irodakhon Rasulova, Mazin A. A. Najm, Munther Abosoda, Ali Alsalamy, Mahamedha Deorari

**Affiliations:** 1 Department of Clinical Laboratory Sciences, College of Applied Medical Sciences, Jouf University, Sakaka-72388, Aljouf, Saudi Arabia; 2 Department of Prosthetic Dentistry, I.M. Sechenov First Moscow State Medical University (Sechenov University), Moscow, Russia; 3 Department of Clinical Laboratory Sciences, College of Applied Medical Sciences, King Khalid University, Abha, Saudi Arabia; 4 Doctor en Gestión Pública y Gobernabilidad, Docente en La Universidad Norbert Wiener, Lima, Perú; 5 Escuela de Posgrado, Universidad San Ignacio de Loyola, Lima, Peru; 6 School of Humanities, Natural & Social Sciences, New Uzbekistan University, 54 Mustaqillik Ave, 100007 Tashkent, Uzbekistan; 7 Department of Public Health, Tashkent Pediatric Medical Institute, Bogishamol Street 223, Tashkent, Uzbekistan; 8 Pharmaceutical Chemistry Department, College of Pharmacy, Al-Ayen University, Thi-Qar, Nasiriyah, Iraq; 9 College of Pharmacy, the Islamic University, Najaf, Iraq; 10 College of Pharmacy, the Islamic University of Al Diwaniyah, Al Diwaniyah, Iraq; 11 College of Pharmacy, the Islamic University of Babylon, Hillah, Iraq; 12 College of Pharmacy, Imam Ja’afar Al‐Sadiq University, Al‐Muthanna, 66002 Iraq; 13 Uttaranchal Institute of Pharmaceutical Sciences, Uttaranchal University, Dehradun, India

**Keywords:** Oropharyngeal Cancer, HPV, Molecular Pathways, Carcinogenesis, Oncoproteins

## Abstract

Oropharyngeal cancer, a subset of head and neck cancer, is increasingly recognized as a unique clinical entity primarily influenced by high-risk human papillomavirus (HPV) infections, particularly HPV-16. This review delves into the viral life cycle of HPV-16 and its interactions with host cells, with a specific focus on the crucial roles played by the viral oncoproteins E6 and E7. These oncoproteins drive cellular proliferation by targeting critical tumor suppressor proteins like p53 and Rb, resulting in uncontrolled cell growth and genomic instability. Furthermore, the significance of epigenetic modifications induced by HPV-16 and their implications is important for cancer progression. This comprehensive review provides valuable insights into the intricate molecular landscape of HPV-induced oropharyngeal cancer, shedding light on the development of targeted therapies and preventive strategies for this emerging global health concern.

Video Abstract

**Supplementary Information:**

The online version contains supplementary material available at 10.1186/s12964-023-01365-0.

## Introduction

In the domain of head and neck squamous cell carcinoma (SCC), oropharyngeal squamous cell carcinoma (OPSCC), often referred to as throat or tonsil cancer, is distinguished by its involvement of the base and posterior third of the tonsils, tongue, soft palate, as well as the posterior and lateral pharyngeal walls [1]. A troubling rise in the prevalence of OPSCC is being observed in both older and younger age groups [2]. OPSCCs has long been associated with alcohol and tobacco consumption, as have head and neck SCCs. However, a significant decline in smoking prevalence in high-income countries over the past two decades has caused a reduction in the incidence of head and neck SCCs [3, 4]. OPSCC can be induced by tobacco, a significant risk factor, which can cause epigenetic changes in oral epithelial cells, suppression of various systemic immune functions of the host, and the generation of oxidative stress on tissues through its toxic metabolites, all contributing to the development of OPSCC. Furthermore, the pathogenesis of OPSCC is believed to be influenced by specific viruses like EBV and human papillomavirus (HPV) [5, 6]. Despite this, during the same period, there has been an alarming increase in the incidence of OPSCC, which is largely attributed to carcinogenic HPV infection [6]. Notably, a substantial proportion of OPSCC cases, representing 51.8% in the UK and 71% in the USA, are now accounted for by HPV infection [7]. Among these cases, the vast majority, about 85–96%, are driven by HPV-16 infections, suggesting a potential for prevention through prophylactic HPV vaccination, which has already demonstrated efficacy against HPV -associated cervical neoplasia and is currently being administered to both male and female individuals in multiple countries [7, 8]. Despite limited knowledge regarding the precise biological mechanisms through which OPSCC is induced by HPV, substantial epidemiological evidence supports an important association between HPV infection and the incidence of OPSCC. Within this review, an extensive overview of HPV-positive OPSCC and molecular mechanisms is provided.

### Human papillomavirus

HPV, as a DNA virus with a diameter of about 55 nm, lacking an envelope is known for its tropism to epithelial cells. It contains a double-stranded DNA molecule, which encompasses about 8,000 base pairs (8 kbp) [9]. The viral genetic material is partitioned into three distinct segments: an early (E) section, a late (L) segment and a lengthy control region (LCR). The E region consists of eight genes, and the L region contains two genes. These genes are responsible for the production of viral proteins, whereas the LCR functions as an upstream non-coding regulatory area, accommodating the site for the initiation of viral DNA replication and transcriptional regulatory elements [10].

Currently, more than 200 different genotypes of *papillomaviridiae* have been identified, exhibiting a minimum of 10% nucleotide variation within the capsid gene (L1) [11]. Several methods have facilitated the categorization of these genotypes by analyzing commonalities within their DNA sequences. They can be categorized as mucosal, primarily belonging to the alpha genus, or cutaneous, mainly falling within the beta genus, based on their preference for specific types of epithelial tissue [11, 12]. Furthermore, they are classified into high-risk and low-risk categories according to their capacity to trigger malignant changes in host cells. Examples of high-risk viruses include HPV 16, 18, 31, and 33, which are identifiable in cases of high-grade squamous intraepithelial lesions in the cervix or invasive cancer [13, 14]. On the other hand, HPVs 6, 11, 40, and 42 are considered low risk viruses and are isolated from mild epithelial lesions of the cervix [15]. Several HPV types pose a potential high-risk factor with uncertain oncogenic potential. Moreover, variability within the same HPV type exists, which may also be associated with disease development, along with regional differences in prevalence of genotype [16]. HPV is acknowledged as a highly potent human carcinogen. It exerts its oncogenic potential through the expression of E6 and E7 genes, which generate the oncoproteins E6 and E7. These oncoproteins inhibit the functions of p53 and Rb proteins, respectively, thereby conferring oncogenic properties to the virus [17]. E6 also interacts with various PDZ domain-containing proteins, contributing to the disruption of cellular adhesion and polarity. Additionally, E7 interacts with a range of other cellular proteins, including cyclins, cyclin-dependent kinases, and histone deacetylases, facilitating the viral replication and cellular transformation necessary for HPV-associated tumorigenesis. The precise interactions with these cellular proteins highlight the multifaceted roles of E6 and E7 in the development of HPV-induced cancers [8–11]. Other HPV early genes, including E5, E4, E1, and E2, serve critical functions in the virus's life cycle. E5 promotes cell growth by activating growth factor receptors, enhancing proliferation, and inhibiting apoptosis, aiding viral replication and immune evasion. E4 facilitates the formation and release of viral particles by disrupting the cellular cytoskeleton, aiding HPV spread. E1 is essential for initiating viral DNA replication, while E2 regulates viral gene expression and genome maintenance, ensuring successful infection and replication. Together, these early genes play crucial roles in the HPV life cycle [8].

### Current hpv detection methods

Current HPV detection methods have undergone significant advancements, leading to improved accuracy and efficiency. These methods encompass Nucleic Acid Amplification Tests (NAATs), including PCR and digital PCR, recognized as the gold standard, with a focus on quantifying viral load. Hybrid Capture Assays, Next-Generation Sequencing (NGS), and the combination of Pap smear and HPV DNA testing for heightened sensitivity are prominent trends. Additionally, immunohistochemistry identifies viral oncoproteins in tissue samples, while the development of point-of-care and saliva-based tests aims to increase accessibility. Ongoing research explores novel biomarkers such as microRNAs and DNA methylation patterns for enhanced early detection. Quantitative PCR and HPV genotyping, achieved through multiplex PCR assays and DNA microarrays, continue to play a crucial role in assessing risks associated with HPV infection, ultimately contributing to the prevention and early diagnosis of HPV-related cancers [8, 11, 17].

### Prevalence of human papillomavirus^+^oropharyngeal SCC

Since the beginning of the twenty-first century, a new type of head and neck cancer, HPV + OPSCC, has emerged [18]. Among all cancers, OPSCC is noted for its rapidly increasing incidence in high-income countries [19]. The disease's prevalence has been on the rise in various regions, including Europe, the UK, New Zealand, USA, and Asia. Importantly, in both the United Kingdom and the United States, the occurrence rate has seen a rise in oropharyngeal cancer in men, surpassing the rates of cervical cancer in women. [19, 20]. The most prevalent oncogenic, HPV genotypes detected in cervical cancer are listed as follows: 16, 18, 31, and 33 [13, 14]. However, there is a variation in the distribution of HPV types between oropharyngeal and cervical cancers [21]. In 2021, approximately 33% of OPSCC were reported to be HPV-positive. However, the prevalence varies significantly across different geographical regions, ranging from 0%-85% in India to Lebanon, respectively [22].

Based on the reports, in sub-Saharan Africa, the incidence of HPV-positive OPSCC seems to be relatively low, with only a limited number of reported cases so far, despite the region having a high prevalence of HPV-associated cervical cancer [19]. In a study into HPV + OPSCC in Mozambique revealed a low prevalence of 14.5% in their cohort, and the authors suggested that this might be attributed to the limited practice of oral sex in the region [23]. Other researchers have also substantiated this finding, as they identified lower rates of oral HPV infection among HIV-infected individuals in Cameroon. They partially attributed this phenomenon to the reduced engagement in oral sexual behaviors compared to higher-income countries [18]. The general population's prevalence of high-risk oral HPV infection is estimated to be around 3.5%-3.7%. However, individuals co-infected with HIV have higher rates of oral high-risk HPV infection [24].

### Risk factors of oropharygeal SCC

In the past, there has been a robust correlation has existed between head and neck SCCs, including those affecting the oropharynx, and individuals with an extended record of substantial smoking and alcohol intake [25, 26]. Prior research have shown that the development of these cancers is directly related to the frequency and duration of tobacco and alcohol exposure, with a clear dose–response relationship [27–29]. Typically, traditional HPV-negative OPSCC typically manifest in individuals in their seventies [30]. Additional factors that elevate the risk of these tumors encompass inadequate oral hygiene, a diet lacking in fruits and vegetables, and persistent inflammatory conditions in the oral cavity [31].

The evidence concerning the role of tobacco and alcohol in oral HPV infection and HPV-related OPSCCs remains inconclusive [32]. While certain studies propose a positive correlation, others indicate no association [33]. In cervical cancer, tobacco smoking is definitely a contributing factor, but this weakens after taking into account sexual and reproductive factors [34]. The evidence concerning the role of tobacco and alcohol in HPV-related OPSCCs and oral HPV infection remains inconclusive. While certain studies propose a positive correlation, others indicate no association [35, 36]. Nonetheless, HPV-related OPSCCs may develop in individuals, regardless of whether they have a history of tobacco and alcohol use or not [37]. The exact contribution of tobacco exposure to the development of HPV-related OPSCCs remains uncertain, although it could potentially amplify the effects of HPV-related carcinogenesis [38]. Some studies have linked marijuana use to OPSCCs [39–41], however, this correlation diminishes when accounting for variables related to sexual behavior.

HPV-related OPSCCs are strongly linked to sexual behaviors, which are correlated with the disease [42]. Numerous studies have demonstrated strong connections, especially in comparison to other head and neck squamous cell carcinoma (HNSCC), between both HPV-positive HNSCCs and OPSCCs and various factors [43]. These determinants encompass various factors, such as the cumulative count of sexual partners over one's lifetime, participation in vaginal, anal, and oral sexual practices, early commencement of sexual activity, previous sexual encounters at a young age, and a history of sexually transmitted infections, including genital warts [44]. However, after adjusting for HPV-16 serology, these correlations no longer maintain their statistical significance. This suggests that sexual behaviors can be considered as proxies for HPV-16 exposure [45]. Data from multiple developed countries have indicated an increasing pattern in markers of high-risk sexual behaviors within recent birth cohorts. These markers encompass the earlier initiation of sexual activity, engaging in premarital sex, a higher average number of lifetime partners, and involvement in oral sexual activities [46].

HPV-positive OPSCCs represent a distinct subgroup that tends to be diagnosed at a younger age, typically several years earlier than HPV-negative tumors [47]. Despite their resemblance to tumors in older patients, HNSSCs occurring in younger individuals display notable genetic distinctions, encompassing both germline and somatic variation [48, 49]. As indicated by a study, individuals under the age of 55 had a 3.4-fold greater risk of carcinogenic HPV infection [50]. Furthermore, a robust connection has been established between HPV-16 infection and Cancer of the tonsils occurring in men below 40 years of age [51]. There is a noticeable surge in the incidence of OPSCC among individuals younger than 60 years old [52], with a particularly steep increase observed in those between the ages of 50 and 59 [53]. Nonetheless, it is plausible that other risk factor exposures specific to this particular birth cohort may contribute to this trend.

HPV-related OPSCCs have a strong association with sexual behaviors, which are linked to the disease [30]. Multiple studies have demonstrated significant connections between both HPV-positive HNSCCs and OPSCCs, when compared to other HNSCCs, and factors like the lifetime number of sexual partners, participation in oral, vaginal, and anal sexual activities, early initiation of sexual activity, or earlier sexual contact, as well as a history of sexually transmitted diseases, including genital warts [36, 54, 55]. However, when adjusting for HPV-16 serology, the associations in a case–control series no longer reached statistical significance, suggesting that sexual behaviors can be considered an indicator of HPV-16 exposure [56]. Information gleaned from various developed countries suggests that recent birth cohorts have exhibited a rise in markers associated with high-risk sexual behaviors. These markers encompass younger ages of sexual initiation, engagement in premarital sexual activity, an increase in the average number of lifetime partners, and participation in oral sex [57–59].

Both non-HPV-related and HPV-related HNSCCs show a higher incidence in males, with a ratio of about 3 to 1 [10]. In the case of alcohol and tobacco-related HNSCCs, this gender gap has diminished over time due to shifting smoking patterns [60]. In 1974, 43% of men and 30% of women smoked, compared to 26% of men and 21% of women in 2000 [61]. Nevertheless, the male predominance persists for HPV-related HNSCC, and the underlying cause remains uncertain. The difference in gender regarding oropharyngeal cancer incidence cannot be solely attributed to variations in sexual behaviors, indicating the existence of potential biological distinctions between men and women [7, 10]. It is also plausible that certain male-specific characteristics may predispose them to this form of cancer [62]. Suggestions have arisen regarding hormonal variances or the possibility of protective immunity generated through seroconversion as a response to cervical infections of HPV in women may play a role [63, 64]. Though there is not unanimous agreement among all studies, the majority of them indicate a higher prevalence of oral infection of HPV in men when compared to women [65, 66]. Furthermore, there is a proposition that the transmission of oral HPV may be more efficient when men engage in oral sex with women, possibly because of a higher HPV copy number present in the vagina/cervix [67]. Oral HPV is primarily contracted through sexual transmission, and its prevalence is linked to particular sexual practices. Research indicates an increase in HPV acquisition during the period surrounding sexual debut [68]. The reported prevalence of oral HPV is 1.5% among 12–15-year-olds, 3.3% among 16–20-year-olds, and 4.5%-6.9% among healthy adults [69]. Elevated oral HPV prevalence is noted in females with cervical HPV infection and individuals living with HIV [70, 71]. While studies have documented consistent oral HPV infections among partners [72], the preliminary findings from the HPV oral transmission study in partners over time do not support these conclusions.

There have been indications that non-sexual HPV transmission may be feasible through actions like kissing, transmission during childbirth, and transmission during laser surgery [73, 74]. Oral HPV-16 infection is viewed as a notable risk factor for oropharyngeal cancer, although the connection is more complex in the case of oral SCCs [75]. However, it is important to highlight that the prevalence of oral HPV is lower than that of cervical HPV, potentially due to a smaller proportion of oral-genital partnerships in comparison to genital-genital partnerships [34]. Nonetheless, the course of HPV infection in the oral cavity seems to parallel that of cervical infections [76]. While the degree of type-specific agreement is limited, it is important to acknowledge that HPV infection in both the cervix and oral cavity is not entirely unrelated [77]. This implies that cervical HPV infection may potentially act as a contributing factor to HPV infection in the oral cavity. The entire progression of HPV infection in the oral cavity and oropharynx is still not entirely comprehended [78]. Nonetheless, it is approximated to have an annual occurrence of 4.4%, with the majority of infections resolving within a year. Nevertheless, alterations in sexual behaviors might lead to increased infection rates, potentially resulting in infections that are more resistant to immune responses.

### HPV Vaccination & oropharyngeal SCC

Enhancing cancer prevention is crucial to align with shifting societal norms. Presently, no early screening techniques exist for OPSCC, emphasizing the significance of widespread vaccination initiatives for prevention [79]. The effectiveness of HPV vaccination in reducing cervical cancer rates has already been demonstrated in high-income countries where it is readily available for girls [80]. Nevertheless, the full elimination of HPV-related cancer risks in men may not be achieved through universal vaccination for girls alone [81]. This is especially relevant for men who have sex with men and those residing in regions lacking comprehensive vaccination programs for girls.

Nationwide vaccination initiatives that encompass boys have been expanded in several countries, including Australia, Italy, Germany, Austria, New Zealand, UK, and the USA [82]. Despite these efforts, challenges to vaccination persist, including concerns related to vaccine safety, socioeconomic factors, and insufficient awareness levels. The dissemination of information and education by healthcare providers can have a substantial impact on vaccine acceptance. Evidence indicates that vaccination has the potential to effectively prevent OPSCC, with HPV vaccination demonstrating its efficacy against HPV infections [83]. Nevertheless, it may require some time for the complete advantages of widespread gender-neutral vaccination to become evident. Consequently, it is anticipated that rates of HPV + OPSCC will increase in the coming decades before significant reductions are observed [84]. In light of this, it becomes imperative to invest in public awareness campaigns and early detection strategies. These measures are crucial for mitigating the substantial human and societal costs associated with HPV-related cancers [85].

### HPV Molecular mechanism in OSCC

HPVs have genetic material composed of circular double-stranded DNA, which is approximately 8000 base pairs long [8]. A total of over 200 HPV types have been recognized, and their life cycles are completed within either cutaneous or mucosal epithelia [86]. Among these types, 14 mucosal HPV types are classified as high-risk by the WHO, including HPV-16, 18, 31, and 33. Both epidemiological and experimental evidence support the notion that these genotypes are linked to cancer causation, with approximately 85% of all [86, 87].

The intricate relationship between HPV-16's productive life cycle and the differentiation of keratinocytes within stratified mucosal epithelia contributes to the process of carcinogenesis [88, 89]. This development commonly occurs within the context of persistent infection, often facilitated by the immune-protected surroundings of tonsillar crypts. It arises from a departure from productive viral replication. This process entails gradual modifications in both viral and host gene expressions, as well as adjustments to the host genome. These alterations have been extensively examined in the context of cervical cancer [90].

The field of understanding how HPV interacts with cellular differentiation pathways has recently witnessed significant activity. In the context of HPV-induced carcinogenesis, a pivotal phase is marked by the activation of two viral early genes: HPV oncogenes E6 and E7 [91, 92]. These genes play a central role in kickstarting the cell cycle within the basal layer of the epithelium, subsequently enabling viral genome replication [92]. It is noteworthy that heightened expression of E6 and E7 is often linked to the integration of high-risk HPV DNA into the host genome, though it's crucial to recognize that carcinogenesis can still occur even in the absence of such integration. As research in this area continues to progress, the dynamic interplay between HPV and cellular differentiation pathways remains a focal point of investigation, shedding light on the intricate mechanisms underpinning HPV-associated malignancies [93]. The interruption of another viral gene, E2, which ordinarily acts to suppress the expression of E6 and E7 during productive infection, is commonly identified in OPSCCs marked by integrated HPV and has been associated with an adverse prognosis [94].

The physical state of the HPV genome holds clinical importance in individuals with HPV + OPSCC [95]. An examination of samples from 84 patients demonstrated that those exhibiting evidence of integrated HPV gene expression experienced shorter overall survival (OS) and reduced antitumor immunity compared to those lacking integration evidence [7]. Extensive studies have been carried out by researchers to comprehend the molecular mechanisms through which E6 and E7 initiate cell-cycle entry and DNA replication within host cells [96, 97]. These processes, combined with modifications to the host genome, may lead to the malignant transformation of the host cell, thereby facilitating the development of numerous cancer characteristics. E6 and E7 exhibit two extensively studied oncogenic activities: enhanced degradation of p53 by E6 and Rb by E7 [98]. This depletion of crucial tumor suppressor proteins leads to the loss of cell-cycle checkpoint activation when DNA damage occurs and allows uncontrolled DNA replication. Consequently, this fosters genomic instability and prevents apoptosis, ultimately contributing to the onset of cancer.

**Fig. 1:**
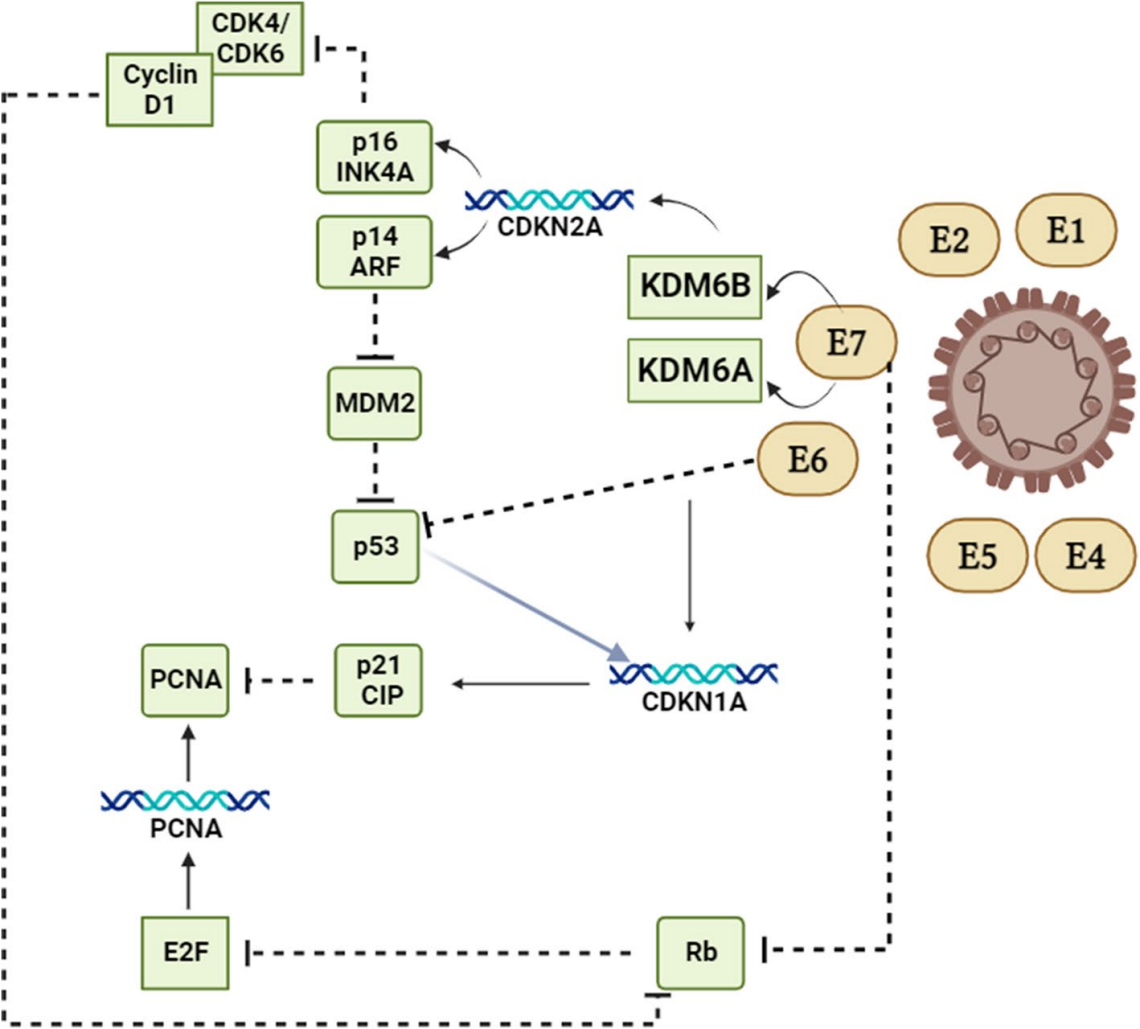
A schematic model of the molecular mechanism behind the disruption of the normal cell cycle process caused by the oncogenic HPV genes E6 and E7

For an extended period, the disruption of Rb function has been acknowledged as a vital oncogenic trait stemming from HPV-induced epigenetic reshaping [99, 100]. This process entails the activation of two lysine demethylases, KDM6B and KDM6A, which function independently of Rb [101]. These enzymes exert a widespread influence on gene expression, including the activation of Homeobox (HOX) genes. HOX genes play crucial roles in developmental regulation, usually under the control of polycomb group (PcG) proteins when there is no high-risk HPV infection present [101, 102]. Furthermore, HPV exerts an influence on chromatin states and alters DNA methylation patterns through E6-mediated regulation of microRNAs and other non-coding RNAs [103]. It is suggested that E7's suppression of Rb function prevents the senescence-like response induced by oncogenes during cellular reprogramming, making HPV-transformed cells reliant on the continual expression of HPV oncogenes. Inhibiting E6 and/or E7 proteins as a therapeutic strategy has posed challenges due to their absence of inherent enzymatic activity [104, 105]. However, there has been progress in leveraging HPV oncoproteins as candidates for therapeutic vaccines. Furthermore, the epigenetic reprogramming in cells transformed by HPV leads to a reliance on the p16INK4A tumor suppressor protein (p16) through the E7-KDM6B axis [7]. This stands in contrast to numerous other types of tumors, where the inhibition of CDK4/6 proves to be an efficacious therapeutic approach. The reliance on p16 underscores the cellular reconfiguration induced by HPV and its critical role in tailored therapeutic approaches for HPV + disease [106, 107]. Moreover, in order to curb the pace of DNA replication and avert replication-related stress, the induction of p21CIP1 expression, governed by CDKN1A and mediated by KDM6A, becomes essential within the context of E7-directed epigenetic reprogramming (Fig. 1) [108, 109].

E6 and E7-mediated rewiring of cell-cycle control suggests that the increase in p16 expression observed in HPV + cancers is a consequence of E7-induced KDM6B, rather than Rb inhibition, which has been widely believed [105]. Furthermore, recent research has shed light on other several intriguing molecular signaling pathways. For instance, the PI3K/AKT/mTOR pathway has been identified as a key player, showing that HPV oncoproteins can activate this pathway, promoting cell survival and growth. Additionally, the Notch signaling pathway has been found to be perturbed in HPV-positive oropharyngeal cancers, influencing cell differentiation and proliferation. Moreover, emerging evidence suggests a connection between the Wnt/β-catenin pathway and the development of these cancers, with interactions involving viral oncoproteins. These pathways, along with previously known ones like the p53 and Rb pathways, collectively contribute to the molecular landscape of HPV-induced oropharyngeal cancer [102–105].

## Conclusion

The biological profile of oropharyngeal cancer associated with HPV infection involves the degradation of p53, the inactivation of the Rb pathway, and an increase in p16 expression. On the other hand, tobacco-related oropharyngeal cancer is characterized by mutations in p53 and a decrease in CDKN2A expression. In contrast, HPV-positive oropharyngeal cancer demonstrates enhanced sensitivity to both chemotherapy and radiation therapies when compared to HPV-negative forms of the disease. The detection of HPV-16 can be regarded as a prognostic indicator linked to better overall and disease-free survival outcomes. However, its utility as a predictive marker remains to be firmly established. Numerous inquiries into the natural progression of oral HPV infection are currently subjects of ongoing research. Concerning disease management, it is acknowledged that HPV-positive oropharyngeal cancer represents a unique category within head and neck SCC, characterized by a more favorable prognosis. Individuals diagnosed with HPV-positive oropharyngeal cancer typically belong to a younger age group and generally exhibit better overall health. In forthcoming clinical trials, categorizing patients with head and neck cancer based on their HPV status is advisable. Regardless of the chosen treatment strategy, there exists an opportunity to investigate less aggressive treatment approaches that preserve survival rates while minimizing the potential for severe side effects.
